# Supplementary material for: Rapid fabrication of hydrogel micropatterns by projection stereolithography for studying self-organized developmental patterning
Source: PLoS One. 2021 Jun 2;16(6):e0245634. doi: 10.1371/journal.pone.0245634 (PMC8172057; doi:10.1371/journal.pone.0245634)
Supplement: S1 Table — (DOCX) [file pone.0245634.s001.docx]

**﻿Supporting information**

**S1 Table. Key Resource Table.**

| **﻿REAGENT or RESOURCE** | **SOURCE** | **IDENTIFIER** |
| --- | --- | --- |
| ***Glass-bottomed petri dish or slice*** | | |
| 35 mm Glass bottom dish with 10 mm micro-well #1.5 cover glass | Cellvis | D35-10-1.5-N |
| 35 mm Glass bottom dish with 14 mm micro-well #1.5 cover glass | Cellvis | D35-14-1.5-N |
| µ-Slide 18 Well glass bottom – Glass coverslip No. 1.5H, selected quality, 170 µm +/- 5 µm | Ibidi Inc | 81817 |
| µ-Slide 8 Well glass bottom – Glass coverslip No. 1.5H, selected quality, 170 µm +/- 5 µm | Ibidi Inc | 80827 |
| ***Chemicals, Peptide, and Recombinant Proteins*** | | |
| Ethanol ( ≥ 190 proof) | - | - |
| Alconox® detergent | Sigma-Aldrich | 242985 |
| Acetic acid | Sigma-Aldrich | A6283 |
| 3-trimethoxysilyl-propyl-methacrylate (TPM) | Sigma-Aldrich | 440159 |
| FluoSpheres™ Polystyrene Microspheres, 1.0 µm, yellow-green fluorescent (505/515), for tracer studies | Thermo Fisher Scientific | F13081 |
| ﻿DAPI (4,6-diamidino-2-phenylindole, dihydrochloride) | Thermo Fisher Scientific | ﻿Cat#D1306 |
| ﻿Human BMP4 Recombinant Protein | ﻿ Thermo Fisher Scientific | ﻿Cat#314BP050 |
| ﻿SB431542 | ﻿Stemgent | ﻿Cat#04-0010-05 |
| ﻿IWP2 | ﻿Stemgent | ﻿Cat#04-0034 |
| Human recombinant laminin-521 protein | Biolamina | Cat# ﻿R021599/X0086842 |
| ﻿Dulbecco's PBS without calcium and magnesium | ﻿Caisson Labs | ﻿Cat# PBL01-6X500ML |
| Dulbecco's PBS with calcium and magnesium | Caisson Labs | Cat# PBL02-6X500ML |
| ﻿mTeSR1 | ﻿STEMCELL Technologies | ﻿Cat#85875 |
| ﻿N-2 Supplement (100x) | ﻿Life Technologies | ﻿Cat# 17502048 |
| ﻿B-27 Supplement (50x), minus vitamin A | ﻿Life Technologies | ﻿Cat# 12587010 |
| ﻿β-Mercaptoethanol | Thermo Fisher Scientific | ﻿Cat# 21985023 |
| DMEM/F12 | VWR | ﻿Cat# 45000-344 |
| ﻿Accutase | Thermo Fisher Scientific | ﻿NC9839010 |
| ROCK inhibitor Y-27632 | Thermo Fisher Scientific | ﻿Cat#50-175-998 |
| ﻿Propidium iodide – 1.0 mg/mL solution in water | Invitrogen | P3566 |
| Calcein-AM in LIVE/DEAD™ Viability/Cytotoxicity Kit, for mammalian cells | Invitrogen | L3224 |
| ***Antibodies*** | | |
| ﻿Rabbit Anti-SOX2 (1:200) | Cell Signaling Technologies | Cat# 5024S |
| ﻿Mouse Anti-TFAP2α (1:100) | Developmental Studies Hybridoma Bank | Antibody Registry ID# ﻿AB2313948 |
| ﻿Goat Anti-BRACHYURY (1:300) | ﻿R&D Systems | ﻿Cat# AF2085 |
| ﻿Mouse Anti-ISL1 (1:75) | Developmental Studies Hybridoma Bank | Antibody Registry ID# AB2314683 |
| ﻿Goat Anti-SOX9 (1:100) | R&D Systems | ﻿Cat# AF3075 Goat |
| ﻿Rabbit Anti-PAX6 (1:300) | Biolegend | Cat# 901301 |
| Mouse Anti Ki-67 (1:50) | DSHB | AFFN-KI67-3E6 |
| ***Experimental Models: Cell Lines*** | | |
| ﻿ESI-017 | ﻿ESI BIO | ﻿RRID:CVCL_B854 |
| ﻿ESI-017 NODAL knockout cells | (6) |  |
| **﻿*Software and Algorithms*** | | |
| MATLAB |  | ﻿https://www.mathwo rks.com/products/matlab.html |
| ilastik | (18) | http://ilastik.org/ |
| FIJI | (21) | https://fiji.sc/ |
