# Supplementary material for: Rapid fabrication of hydrogel micropatterns by projection stereolithography for studying self-organized developmental patterning
Source: PLoS One. 2021 Jun 2;16(6):e0245634. doi: 10.1371/journal.pone.0245634 (PMC8172057; doi:10.1371/journal.pone.0245634)
Supplement: Attachment — Submitted filename: Response_0413_clean.pdf [file pone.0245634.s002.pdf]

We appreciate the time and effort that the reviewers have dedicated to providing valuable feedback on our manuscript, and are grateful for their constructive criticism. We have now revised the manuscript to reflect most of the suggestions provided by the reviewers, and feel that it is significantly improved. We have highlighted the changes within the manuscript, and a point-by-point response to the reviewers' comments and concerns appears below.

Comments from Reviewer #1:

Comment 1: *The Measurement of gel thickness is demonstrated in figure 3a, but why not show the SEM or bright filed image of the increasing thickness of the patterned gel? e.g., labeling the thickness on each image, and the curve size is none necessary to be so large to cover so much space in one figure.*

Thank you for this suggestion. In the case of our study, the micropatterned PEGDA gel bed needs to be hydrated at all times before and during cell culture. However, hydrogels need to be dehydrated for SEM imaging, and the surface morphology of dehydrated gel will differ from the gel surface exposed to the cells in culture. SEM is not an effective method for measuring the thickness of the gel. Furthermore, because the hydrogels are >80 wt% water, the refractive indices of the features and the cell culture media are too similar to adequately assess layer thickness using top-down brightfield microscopy. Thus, we believe that the thickness measurements in our manuscript are more accurate and neither SEM nor brightfield microscopy was performed here for thickness assessment.

**Deleted:** between

Comment 2: *Only the staining results are demonstrated in the results of hESCs culturing, but the cell viability and proliferation rate need to be tested and quantified, which are significant results to demonstrate the biocompatibility of the material and techniques.*

Thank you for this suggestion. Cell viability and proliferation assessments were added to the paper in Figure 4 for a better understanding of the micropatterning method.

Comment 3: *Reproduction of gastrulation and neural ectodermal induction results with the micropatterning method was demonstrated in figure 4. However, it is better to do the marker staining quantification via image analysis, and the marker protein staining results of NODAL-/- in figure 4b is missing, which should also be provided.*

Thank you for this suggestion. The marker staining quantification has been performed and added to the manuscript.

Regarding NODAL-/- cells in Figure 4B, NODAL does not play a role in these ectodermal patterns which are created in the presence of a NODAL inhibitor (SB431542), and therefore we do not expect the phenotype of the NODAL-/- cells to differ. Thus, we only used NODAL-/- cells in the gastrulation patterning assay as

NODAL protein plays an important role in gastrulation, but not in neural ectodermal induction.

Comments from Reviewer #2:

Comment 1: *Some grammar and format errors should be carefully checked through the manuscript.*

Thank you for your suggestions, we have attempted to remove all formatting and grammatical errors

Comment 2: *In the abstract part, the application of this method together with LN521 should be further introduced in more detail.*

Thank you for your suggestion. LN-521 as an extracellular matrix coating for hESC attachment was described in *Nature Methods 2014, 11(8), 3178-3182*, and *Nature Protocols 2016, 11(11), 2223-2232*. PEGDA is resistance to protein adsorption, so the LN-521 can only be coated onto the glass surface, not the PEGDA hydrogel surface. In this way, the hydrogel forces cells to grow in a confined geometry on the LN-521 coated glass surface. We now explain these points more clearly in Discussion and Conclusion on Page 14.

Comment 3: *In the introduction part, this technique has been used by previous scientists, why it can be used to two-dimensional (2D) micropatterning of hydrogel features on glass surfaces using a single virtual photomask, the idea of using the technique is still not especially clear.*

Thank you for your question. SLATE performs the 3D hydrogel printing in a layer-by-layer fashion. In other words, a 3D object is obtained by sequential photopatterning (2D) from the bottom to the top. In the current study for 2D patterns, instead of doing sequential photopatterning through z-direction, a single photopattern on the glass surface is sufficient to produce 2D micropatterns with the geometrical features specified on the virtual photomask.

Either SLATE or the traditional photolithography can be used in generating hydrogel patterns. In either case, a spatial light modifier is needed to generate a 2D pattern of light. Traditional photolithography employs a physical photomask to spatially control these light patterns. This mask is physically manufactured as an opaque sheet with 2D patterns of holes or transparencies. Because each photomask must be separately manufactured, we prefer to use a complete, commercially available system (SLATE) which uses a standard projection system with dynamically tunable photomasks. This allows patterns to be rapidly optimized.

**Deleted:** ,

**Deleted:** physically

Comment 4: *In the introduction part, if the Methacrylation of Glass Surface is different or similar to prevous method, if is so, please give the previous literature.*

Thank you for your suggestion. The methacrylation protocol was used before in Bagrat 2021, which we now cite properly in the revision.

Comment 5: *As for the Characterization of micropatterned PEGDA gel and cell pattern, as for the expression "The thickness of the gel is substantially larger than the height of hESCs grown on a glass surface. Therefore, the micropatterned gel created a well-shaped space for cells to attach and grow." some recent literatures such as , Biomaterials, 2021, 265,120456, Mol. Pharmaceutics 2020, 17, 4, 1300-1309. Chin Chem Lett 2020, 31(12), 3178-3182, ACS Nano, 2020, 14 (10), 13536-13547 should be added.*

Thank you for your suggestion. These papers are about polymer nanoparticles for drug delivery, which is not relevant to our study on micropatterned hydrogel fabrication.

Comment 6: *The stability of gel and cell pattern should be considered.*

Thank you for your suggestion. In the case of our study, PEGDA is a commonly used hydrogel with high biostability in vitro in a short term, like the cell patterning studies. The stability of PEGDA is studied as a control material in *Biomaterials, 2010, 31(13), 3736-3743*. Also, the results of the cell patterns are also presented in the paper, which are comparable to the previous results done with the CYTOO micropatterns. Thus, the stability of gel or cell pattern are not studied here. Additionally, the crosslinking bond forms as an ester, which are typically understood to remain stable in aqueous environments for several weeks if not months. If longer culture periods are needed, the PEGDA can be substituted for PEG diacrylamide, which has an aqueous half-life on the order of years. We now mention these considerations in the discussion section.

Comment 7: *Discussion and Conclusion should be divided into two parts, the discussion is not enough. The comparsivon between the current result and previous work is needed.*

Thank you for this suggestion. The discussion part is extended. In discussion, the fabrication method studied in this paper is compared with the method by Yang et al (2015). Also, the results of self-organized hESC patterning using the micropatterns showed in Figure 4 are also compared to previous work in our lab using the CYTOO micropatterns.

Comment 8: *The reference style should be uniformed.*

Thank you for your suggestion. All references are in Vancouver style following the reference format specified on the PLOS ONE submission guidelines.

Comments from Reviewer #3:

Comment 1: *Why did the authors choose poly(ethylene glycol) diacrylate (PEGDA) as the material of hydrogel?*

Thank you for the question. PEGDA is a commonly used biomaterial, and has been used successfully in other studies in our lab. It has several advantages: biocompatibility, mild reaction conditions, and its known ability to prevent cell adhesion. The crosslinking of PEGDA happen at room temperature with a photo-initiator and light source, which is a mild condition that can reach easily. Specifically, cell-adhesion is limited by PEGDA because the polymer is entirely comprised of short repeating C-C-O units, which lack both peptide and non-specific cell-adhesive moieties. Furthermore, because PEGDA is an extremely hydrophilic polymer, there is little opportunity for the hydrophobic effect to drive protein adsorption, which could otherwise aid in cell adhesion if a hydrophobic substrate was chosen.

Comment 2: *What were the advantages of stereolithography compared to traditional micropatterning methods?*

Thank you for the question. The stereolithography used in our study uses the Lumen X printer, which avoids the use of a photomask or fabrication of a master and a stamp that are required for traditional micropatterning methods like micro-contact printing. The purpose of the study is to develop a fabrication method that is rapid and low-cost, which was discussed in the introduction section. Thus, we prefer to use a complete, commercially available system (SLATE) which uses a standard projection system with dynamically tunable photomasks. This allows patterns to be rapidly optimized.

Comment 3: *The surface morphology of micropatterned surfaces should be characterized by a scanning electron microscope.*

Thank you for your suggestion. As mentioned in previous answers, the micropatterned PEGDA gel bed needs to be hydrated all the time before and during cell culture. However, hydrogels need to be dehydrated for SEM imaging, in which case the surface morphology of dehydrated gel cannot represent the gel surface exposed to the cells in culture. Thus, SEM would not be informative and was not performed here.

Comment 4: *Why did cells not attach to the surface of PEGDA hydrogel?*

Thank you for your question. PEGDA is biologically inert and intrinsically resistant to protein adsorption and cell adhesion. Specifically, cell-adhesion is limited by PEGDA because the polymer is entirely comprised of short repeating C-C-O units, which lack both peptide and non-specific cell-adhesive moieties. Thus, neither cells nor extracellular matrix coating (e.g. LN-521 used in this study) attach to the surface of PEGDA hydrogel.

Comment 5: *What was the mechanism of cell attachment to the LN521-coated glass surface?*

Thank you for your question. LN-521 as an extracellular matrix coating for hESC attachment was described in Rodin et al Nature Communications 5:3195 (2013). As is well known, integrins expressed on the cells bind to laminins on the culture surface. PEGDA is resistance to protein adsorption, so the LN-521 will only be coated onto the glass surface, not the PEGDA hydrogel surface. In this way, the hydrogel forces cells to grow in a confined geometry on LN-521 coated glass surface. Further introduction of LN-521 was included in the revision.

Comment 6: *What were the application prospects of micropatterning surfaces?*

Thank you for your question. In the case of our study, self-organized developmental patterning study is the main application of the micropatterning surfaces. Figure 4 is devoted to these applications. Studies using other cell types that need geometrical confinement can also use this micropatterning surfaces.

Comment 7: *The recently published review and research articles should be discussed in the revision, for example, Nature Methods 2020, 17 (1), 50-54; Journal of Tissue Engineering 2020, 11, 2041731420943839; Cells 2019, 8 (8), 886.*

Thank you for your suggestion. Among the three articles mentioned above, the following two articles are about mesenchymal stem cells for regenerative medicine: *Journal of Tissue Engineering 2020, 11, 2041731420943839; Cells 2019, 8 (8), 886. Nature Methods 2020, 17 (1), 50-54* is about micropatterning of cryo-EM grids for in-cell structural studies. However, none of these studies are relevant to our study which is focused on development of a micropatterning fabrication method and human pluripotent stem cell culturing for developmental biological research.

Comments from Reviewer #4:

Comment 1: *The abstract needs to rewrite. The authors didn't highlight enough their*

*own discoveries. In another word, they discussed too much about the previous stuff, which could reduce the significance of this study.*

We have reviewed the abstract and only the first two sentences explain previous work, while the remainder describes our new results. We believe this is a standard amount of introductory material for an abstract and have therefore chosen to leave it as is.

Comment 2: *Where is the data for characterization of hydrogels? I couldn't get a general idea bout the hydrogel.*

Thank you for your suggestion. PEGDA is a widely used and studied biomaterials. Our study focuses more on the development of the fabrication process and hESC culturing on the micropatterning surfaces. Data on cell viability and proliferation are added to the revision. Regarding hydrogel characterization, in our previous work with the SLATE system (Grigoryan, 2019) we showed that PEGDA hydrogels with >80% water content are able to support high hMSC viability and osteogenic activity. Furthermore, this work showed these gels to have a storage modulus (G') of 30-40 kPa, which is stiffer than typical collagen, matrigel, or fibrin gels (100-1000 Pa). Rather, they are more similar to polyacrylamide gels used for gel electrophoresis.

Comment 3: *The authors claimed that the hydrogel thickness is the limitation in this study. However, there are also some other concerns. For example, the stiffness of the hydrogel may be important for this study, which could affect a lot of stem cell differentiation. Moreover, why the authors only use the blue light projector? The properties of the hydrogel could be tuned with different light sources. Therefore, I suggest the authors try different methods or do more characterizations for the hydrogel following the literature below. At least, the authors need to give some discussion about those questions.*
*(1) DOI: 10.1021/acsami.5b11811*
*(2) DOI: 10.1016/j.cclet.2018.06.009*

Thank you for your suggestions. As we note above, we do not grow cells on the hydrogel but rather on the hydrogel to exclude cells from certain regions of the culture. Thus, the physical properties of the hydrogel are not important for the present study as long as cells do not adhere to it. Blue light is an effective method for polymerization and there is no reason to try other wavelengths.

The article, *DOI: 10.1021/acsami.5b11811*, is about hydrogel development for wound healing. The article, *DOI: 10.1016/j.cclet.2018.06.009*, is about a photocleavable hydrogel for light-triggered drug delivery. Neither of them is relevant to our study on micropatterning fabrication development.

Comments from Reviewer #5:

Self-organized patterning of mammalian embryonic stem cells on micropatterned surfaces has previously been established as an in vitro platform for early mammalian developmental studies, complimentary to in vivo studies. Therefore, the work is interesting and impportant. The authors have proved their idea and thus the manuscript can be published as it is.
